# Supplementary figures and images for: Drosophila R8 photoreceptor cell subtype specification requires hibris
Source: PLoS One. 2020 Oct 14;15(10):e0240451. doi: 10.1371/journal.pone.0240451 (PMC7556441; doi:10.1371/journal.pone.0240451)

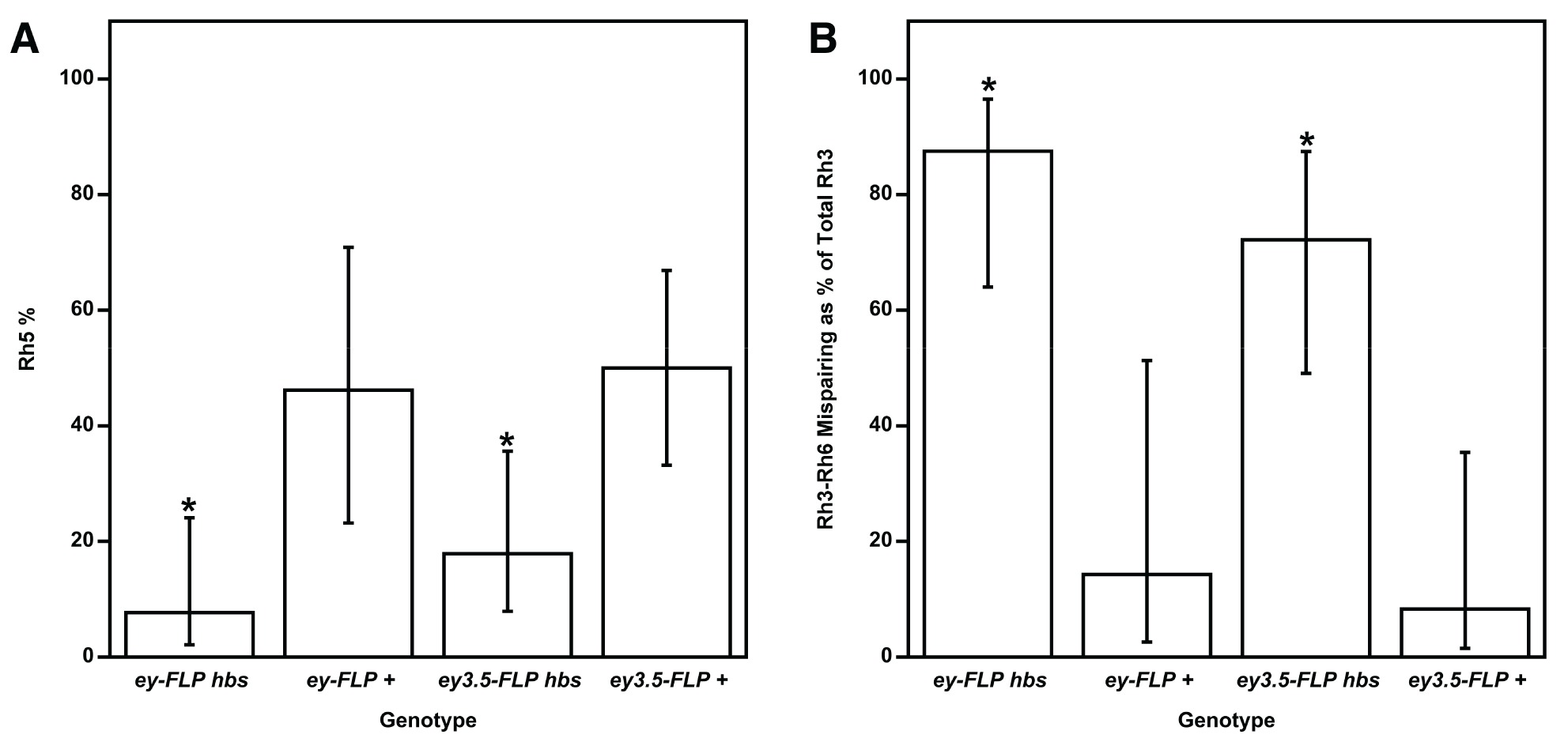

Supplement: S1 Fig — The bar graphs show quantification of the experiment in Fig 7. A Rh5% expression compared to Rh6 is reduced in ey-FLP hbs and eye3.5-FLP hbs (first and third columns from the left), compared to ey-FLP + and eye3.5-FLP + controls (second and fourth columns from the left). B Rh3/Rh6 mispairing % compared to Rh3/Rh5 is increased in ey-FLP hbs and eye3.5-FLP hbs (first and third columns from the left), compared to ey-FLP + and eye3.5-FLP + controls (second and fourth columns from the left). Asterisks indicate p<0.05. Error bars indicate the 95% confidence intervals for the measured percentages. Additional quantitative data in Fig 7 Legend. (TIF) [file pone.0240451.s001.tif]
