# Supplementary material for: Drosophila R8 photoreceptor cell subtype specification requires hibris
Source: PLoS One. 2020 Oct 14;15(10):e0240451. doi: 10.1371/journal.pone.0240451 (PMC7556441; doi:10.1371/journal.pone.0240451)
Supplement: Attachment — Submitted filename: Response to reviewers.odt [file pone.0240451.s006.odt]

Response to reviewers

The reviewer’s comments are reproduced below with our point by point response.

Reviewer #1: In this paper, Tan et al. investigate the molecular basis of R8 subtype specification. They perform a genetic screen for regulators of this process and identify the cell adhesion molecule hbs. Through loss- and gain-of-function experiments, they demonstrate that hbs is necessary and sufficient for Rh5 expression in R8 cells and for appropriate matching of Rh3-expressing R7s and Rh5-expressing R8s. Specific comments and suggestions are below.

Experiments/Interpretation:

Cell Autonomy/ Previous Figure 10 (Current Figure 9): The authors use ey-FLP to generate hbs mutant clones and analyze Rh3-Rh6 mispairing in ommatidia with mutant and wild-type R7 and R8 cells. They observe a high rate of mispairing – almost 60% - in ommatidia containing wild-type or heterozygous R7 and R8 cells and use this observation to argue: “these results unambiguously indicate that hbs is required non-cell autonomously for the establishment of paired opsin gene expression in the R7 and R8 photoreceptor cells.” This conclusion is vastly overstated. An alternative explanation is that the mispairing is due to genetic background effects. I urge the authors to either 1) specifically delete hbs in defined non-R7/R8 cell populations and analyze the effects on Rh3-Rh6 mispairing or 2) remove this figure and argument from the paper.

Author Response: The description of the result has been changed to “The results indicate…” In addition, a more appropriate control has been used, which is designated FLP FRT in Fig 9C left column, w1118 P{ry[+t7.2]=ey-FLP.N}2; P{ry[+t7.2]=neoFRT}42D P{w[+mC]=GMR-myr.GFP}2R This strain contains the same ey-FLP element and is homozygous for the FRT myr.GFP chromosome. Although there is some background effect as suggested by the reviewer, in a hbs66 mosaic mutant ommatidia having homozygous or heterozygous R7 and R8 wild-type cells (R7+ R8+, second column from the left Fig 9c) show a statistically significant increase in the percentage of mispaired Rh3-Rh6 expression (p=0.009) compared to the FLP FRT control. These results, along with those for R7- R8+, and R7+ R8- ommatidia, are consistent with the original conclusion that hbs has non-cell-autonomous effects on opsin expression in the R7 and R8 photoreceptor cells, that cannot be attributed to either the R7 or R8 cell.

Notch/ Previous Figure 11 (Current Figure 10): The authors use a temperature-sensitive allele of N to examine its role in R7-R8 pairing. These experiments seem disconnected from the rest of the study, which is focused on hbs. I recommend removing this figure from the paper.

Author Response: The rationale for the experiments with Notch (N) were poorly described in the original manuscript. Notch is known to regulate hbs during myoblast fusion and controls cell adhesion in the eye through interactions with hbs. In addition, hbs is thought to play a role in N cleavage. For these reasons, N was an obvious additional candidate for analysis in this study because of its interactions with hbs. The rationale for the experiments with N is described more thoroughly in the revised manuscript.

Page 9: The authors describe the genetic background in two contradictory ways. Lines 200-202: “We used the ey-FLP driver to generate homozygous mutant clones in the retina and optic lobes of animals that were heterozygous for hbs1130.” Lines 203-205: “Fig. 7 shows a small heterozygous clone…in an otherwise homozygous mutant retina.” Obviously, the interpretation of this experiment depends on whether the clone is heterozygous or homozygous for the mutant allele.

Author Response: Fig 7 was redundant and has been removed.

Figure references/legends: Both the figure legends and the references to figures in the main text contain many errors. Beginning on page 9, the authors refer to the wrong figures continuously (e.g. on line 211 they refer to Fig. 6A when the data they are describing is actually represented in Fig. 8A). There are also several panels of figures that are never referred to in the main text (e.g. Fig. 8B-C) and panels of figures that are not described in figure legends (e.g. Fig. 2D). The authors must carefully proofread the entire manuscript to ensure that 1) every reference to a figure panel in the main text refers to the intended panel, 2) every figure panel is referred to in the main text, and 3) every figure panel is described in the accompanying figure legend.

Author Response: The text has been carefully edited to remove these errors. 

Figures:

Figure 2: It would be nice to have the quantification of these phenotypes in the figure rather than in a separate table.

Author Response: Quantification is now included in the figure with a referral to Table 1 for details. 

Figure 6: The blue is difficult to see. It should be brightened.

Author Response: Fig 6 has been replaced.

Figure 7: The blue is difficult to see. It should be brightened.

Author Response: Fig 7 has been deleted.

Previous Figure 8 (Current Figure 7): The authors should quantify Rh5 expression frequency and Rh3-Rh6 mismatching frequency in hbs mutant clones.

Author Response: The numbers requested by the reviewer are now included in the figure and with the values and statistics also included in the figure legend. 

Previous Figure 9 (Current Figure 8): The authors should include statistical analyses of the Rh5/Rh6 expression phenotypes.

Author Response: The statistical analysis is included in the figure legend.

Reviewer #2: 1) Comments regarding the results and their interpretation

- The authors claim that a69 is a novel allele of the gene hbs. Even though I agree that a69 is likely to correspond to a novel hbs allele, the authors should provide a direct evidence by demonstrating that expression of hbs is able to rescue the a69 mutation. This is crucial since the complementation pattern between a69, previously identified hbs alleles and deficiencies is not straightforward. In addition, exon sequencing of the hbs gene failed in revealing a genetic lesion unique to the a69 allele. Thus, one cannot exclude that the a69 affects another gene – for instance by targeting a non hbs enhancer.

Author Response: We have not attempted to rescue the a69 mutation by expression of the hbs gene. While a successful rescue experiment would provide additional evidence that a69 is a mutation in hbs, we chose to shift the analysis within the manuscript to the well characterized, bona fide, hbs66 allele. Additional text has been added regarding the caveats noted by the reviewer.

- In the paper, the authors make use of different hbs alleles – some which shown not to complement a69 (e.g. hbs1130) and others that were not tested in complementation with a69 or with deficiencies (e.g. hbs66). The authors should provide more details about the status of these different alleles as well as the logic of using one vs another.

Author Response: As noted above, we chose to focus on the hbs66 allele that has been characterized at a molecular level and is available on a convenient FRT chromosome (42D). Additional complementation experiments with hbs66, a69, other hbs alleles and deficiencies have been performed and these are included in Table 2, and Supporting Information S3 Table. 

- The authors claim that hbs is expressed coordinately with senseless expression in R8s just posterior to the MF. However, the image that is provided (Fig 6) does not allow the reader to conclude about this statement:
o A high magnification inset should be added.
o In addition, the eye disc shown on this figure comes from a very late L3 larva. At this stage, the morphogenetic furrow (MF) has swept across the entire eye field. It is therefore difficult to appreciate whether Hbs is truly only expressed posterior to the MF. One could easily solve this question by staining slightly younger discs (with less advanced MF), which would allow controlling whether hbs is or not expressed anterior to the MF.

Author Response: The figure has been replaced with an image of a younger larva and insets at higher magnification. The text has been modified for clarity.

- Overexpression of hibris: in order to conclude about whether the effect of hbs overexpression is – at least in part – independent from sev, the authors should provide a control demonstrating that all (and if not all, what proportion of?) R7 cells are gone in their sev14 stock.

Author Response: Information regarding the sev14 allele used in the experiment is now included in the figure legend.

- In the discussion (lines 416 and 438), the authors speculate that the expression of hbs in the optic lobes could play a significant role in the induction of Rh5 expression in R8s. However, their results indicate that losing hbs function in the eye alone is sufficient to cause the phenotype. Could the authors be more explicit as to why they still consider a role of hbs in the optic lobe?

Author Response: The text in the Discussion noted above has been deleted.

- Also, in the discussion (lines 433 to 435), the authors comment that their findings that N LOF affects the proportion of Rh3/Rh4 expressing R7s and – at least at some developmental stages- increases the mispairing between Rh3 and Rh6, “raise several notes of caution regarding the potential complexity of the system and the need to rigorously test the underlying hypotheses upon which the current model for R7 and R8 photoreceptor cell differentiation is based”. In my opinion, this sentence is not very clear – do the authors suggest that the current models are not complex enough or at the contrary too complex? I suggest to the authors to rephrase this sentence in order to make their point clearer – in what respect precisely do they consider that their findings cast doubts on current models of R8 cell differentiation?

Author Response: The text in the Discussion noted above has been revised.

2) Minor points
- Scale bars should be added on figures 2, 5, 6, 7, 8, 9, 10
- Citations of the figures in the main text should be edited (the numbers do not always correspond)

Author Response: Scale bars have been added to the figures noted and citations in the text to the figures have been corrected.

- Figure 7: please delineate the heterozygous clone on panels B and C.

Author Response: Fig 7 has been removed.

- Previous Figure 10 (Current Figure 9): colors on the figure and in the legend do not correspond.

Author Response: Colored labels have been added to the figure for clarity.

3) Data availability
Genetic screen: the authors should provide more detailed information – in addition to the genotype of the original P element line - regarding the collection of the 150 eye-expressing lines used in their screen. The authors cite the paper from Callahan and Thomas (1994) in which 1500 lines were generated. Were the 150 eye-expressing lines pre-selected from this collection based on the expression of lacZ in the adult eye? in the eye disc? Is the list of these 150 lines publicly available?

Author Response: The Materials and Methods has been revised to include the generation and screening of the lines noted above. The collection was not retained long term and has since been discarded.
